# Sacral Dysmorphism Increases the Risk of Superior Gluteal Artery Injury in Percutaneous Sacroiliac Joint Fusion: Case Report and Literature Review

**DOI:** 10.7759/cureus.19532

**Published:** 2021-11-13

**Authors:** Garrett Maxwell, Kristopher A Lyon, Lokeshwar S Bhenderu, Garret Schuchart, Ronak Desai

**Affiliations:** 1 Orthopedic Surgery, Baylor Scott & White Medical Center, Temple, USA; 2 Surgery, Texas A&M College of Medicine, Temple, USA; 3 Neurosurgery, Baylor Scott & White Medical Center, Temple, USA

**Keywords:** superior gluteal artery, sacroiliac joint fusion, sacroiliac joint arthrodesis, lumbosacral transitional vertebrae, sacral dysmorphism

## Abstract

Sacroiliac (SI) joint dysfunction is a significant contributor to low back pain. Percutaneous SI joint fusion is a minimally invasive procedure that can provide excellent pain relief for patients, but it is not without complications, especially in patients with abnormal lumbosacral anatomy. We report the case of a 71-year-old man with sacral dysmorphism who had a painful SI joint that was refractory to conservative therapy. After undergoing an elective percutaneous SI joint fusion, he was discharged in stable condition. He returned in a delayed fashion with a large subgluteal hematoma. Imaging revealed disruption of a branch of the superior gluteal artery (SGA). Surgical exploration and ligation of the SGA were undertaken. Sacral dysmorphism affects SI joint fusion procedures by altering sacral anatomy and the safe zones for SI joint implants. Variations in lumbosacral anatomy can also alter the course of the SGA and adjacent nerves. Due to the wide prevalence of sacral dysmorphism, especially in the setting of low back pain, pre-surgical planning to avoid iatrogenic injuries must be considered with advanced imaging studies such as a computed tomography angiogram of the pelvis or catheter-based angiogram, or alternative surgical approaches to the SI joint must be taken.

## Introduction

Pain arising from the sacroiliac (SI) joint is increasingly recognized as a substantial contributor to low back pain. In fact, up to 30% of low back pain can be secondary to dysfunction of the SI joint, especially in the setting of an unrevealing spine survey [1]. Normally, robust ligamentous connections spanning the dorsal aspect of the sacrum and ilium act to limit motion around the SI joint. Many variables can lead to laxity or degenerative changes of these ligaments including pregnancy, increasing age, and long-segment spinal fusions [2]. Ignoring the SI joint in the differential diagnosis of low back pain can be costly. Given the extensive cost of spine surgery, Polly et al. estimate that $3,000 of healthcare spending per patient could be saved if practitioners consider SI joint dysfunction in the differential diagnosis when evaluating a patient presenting with low back pain to avoid unnecessary lumbar spine fusions [3].

Percutaneous SI joint fusion is a quick and relatively simple procedure that provides excellent pain relief in patients suffering from SI joint pain. This minimally invasive procedure is commonly performed with either fluoroscopic or stereotactic navigation assistance to place one or more implants across the SI joint line. However, when a patient has dysmorphic anatomy in the lumbosacral region, there is a risk of injury to blood vessels and nerves during the placement of implants across the joint line [4]. We report here the case of a patient who underwent percutaneous, transgluteal approach SI joint fusion that was complicated by injury to a branch of the superior gluteal artery (SGA) with delayed development of a postoperative hematoma requiring a second operation. We additionally performed a literature review to investigate how the presence of sacral dysmorphism leads to increased risk of vascular and neurological injury in patients undergoing percutaneous SI joint fusion.

## Case presentation

**Figure 1:**
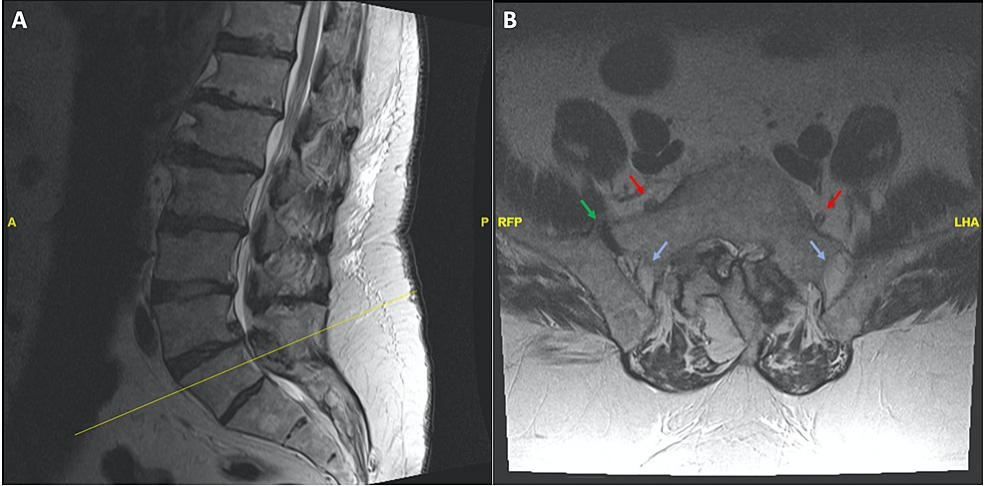
MRI of lumbar spine: (1A) T2-weighted sagittal scan and (1B) T2-weighted axial scan showing L5 nerve roots (red), ligaments (blue), and anterior joint space (green). Incidental hemangioma seen in S2 vertebral body.

A 71-year-old man initially presented to clinic with right hip and low back pain with radiation to his posterior right thigh. He denied numbness, tingling, and weakness of his bilateral lower extremities. He had reproducible pain in the right SI joint region after SI joint provocative testing. A radiographic spine survey was unrevealing for a structural source of low back pain or radiculopathy. Preoperative imaging, including MRI, did reveal lumbarization of the S1 vertebra, mammillary bodies in the alar region, and a residual S1-S2 disc (Figure 1A, 1B). After obtaining adequate pain relief from two separate right-sided SI joint injections, the patient decided to pursue surgical intervention in the form of SI joint fusion using the iFuse implant System® (SI-Bone, Inc., Santa Clara, California).

**Figure 2:**
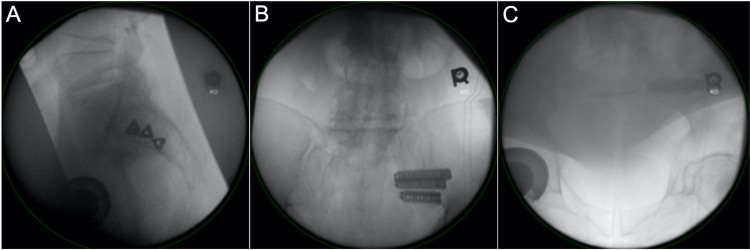
X-ray fluoroscopy of the pelvis showing final implant placement: (2A) lateral view, (2B) an outlet view, and (2C) an inlet view.

In the operating room, the patient underwent general anesthesia and was positioned prone on the Jackson table. He was then prepped and draped in the usual sterile fashion. Fluoroscopy was used to mark the borders of the sacrum on the patient’s skin. Next, a 3 cm incision was made parallel to the sacrum beginning over the center of the S2 vertebral body as approximated with lateral fluoroscopy. The incision was carried down through the gluteal fascia. The first implant was placed within the S2 body caudal to the level of the residual S1-S2 disc on the lateral radiograph. The mid-S2 body was chosen as the site for the first implant in an effort to avoid iatrogenic L5 nerve root damage from starting more cranial due to the known sacral dysmorphism seen on preoperative imaging. The next pin was placed caudal to the first using a double-barrel guide. After the path for the second implant was broached, there was an immediate flow of bright red blood from the wound. The second implant was quickly inserted, and the bleeding stopped. Therefore, we decided to proceed with the third implant (Figure 2). The third implant was then placed, and the wound was irrigated and closed without evidence of continued bleeding. The patient was discharged home the same day in stable condition.

**Figure 3:**
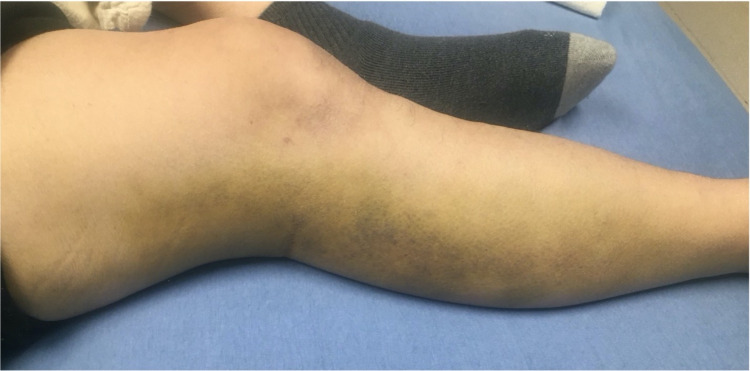
Patient presented with large ecchymosis extending from the right hip to leg on postoperative day 19.

**Figure 4:**
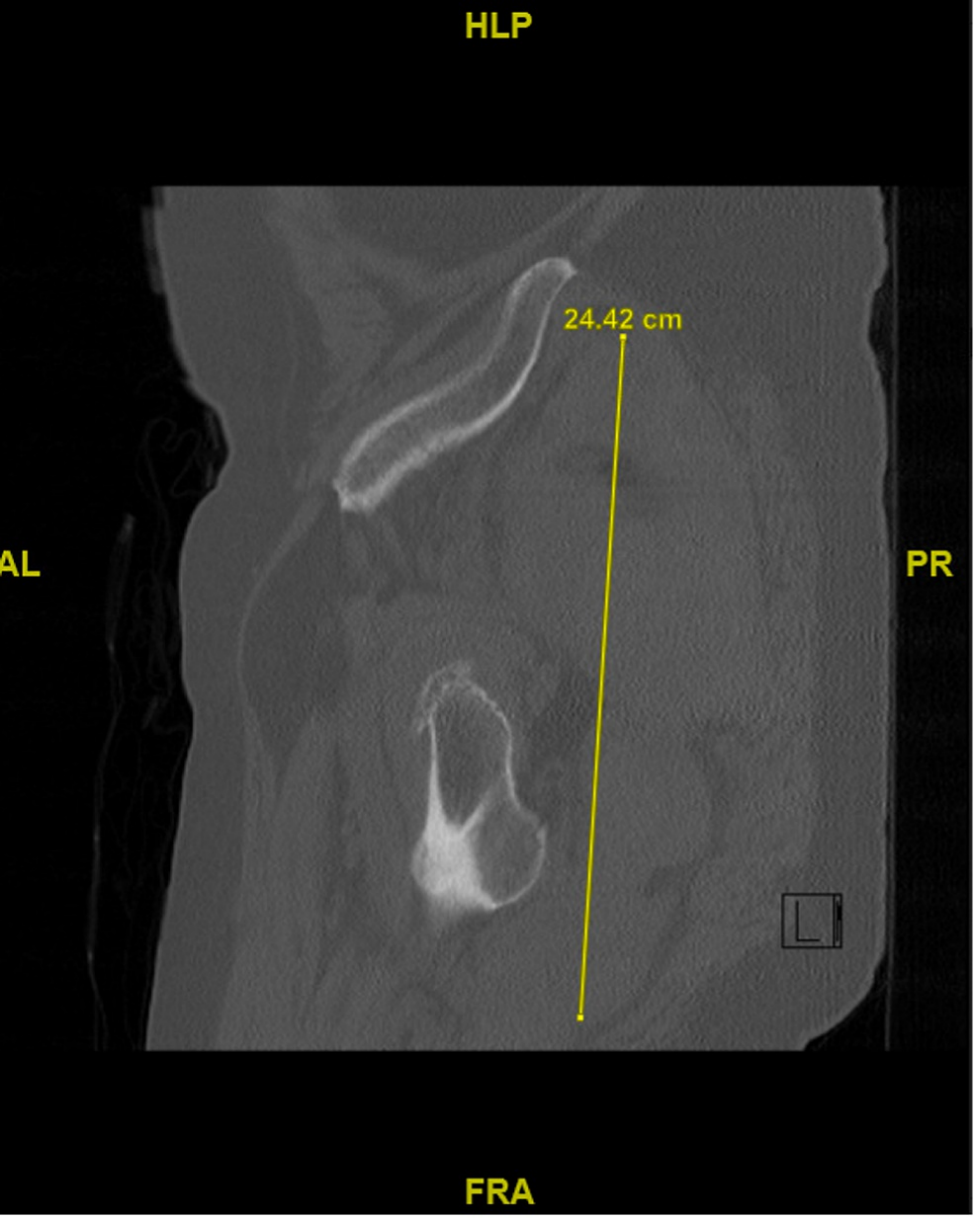
CT of pelvis showing a large intramuscular hematoma, extending inferiorly along the posterior aspect of the femur.

**Figure 5:**
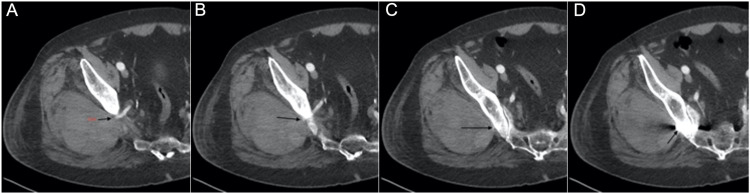
Computed tomography angiography of right lower extremity. A series of axial cuts from caudal to cranial, A to D, showing the superior gluteal artery (black arrow) exiting the greater sciatic foramen and traveling superiorly to end at the side of the implants.

On postoperative day 19, the patient presented to the emergency room with a three-day history of pain in the right gluteal and posterior thigh regions. He also complained of dizziness and occasional syncope when ambulating. Physical exam revealed a well-healed surgical incision with a large bruise extending from his right hip down the right leg (Figure 3). CT of the pelvis showed a large gluteal hematoma with extension into the posterior compartment of the thigh (Figure 4). CT angiogram (CTA) of the right lower extremity showed disruption of the blood flow through the superior gluteal artery at the level of the inferior two implants as the vessel exited the greater sciatic notch (Figure 5). Interventional radiology was consulted for possible embolization, but they determined there was no visible active extravasation and recommended observation and serial hemoglobin draws. The patient’s hemoglobin on the day of presentation was 9.8 g/dL, and on hospital day one it was 8.5 g/dL. Due to this drop in hemoglobin over 24 hours and the patient’s increasing pain level, the decision was made to take the patient to the operating room for exploration and removal of hematoma.

**Figure 6:**
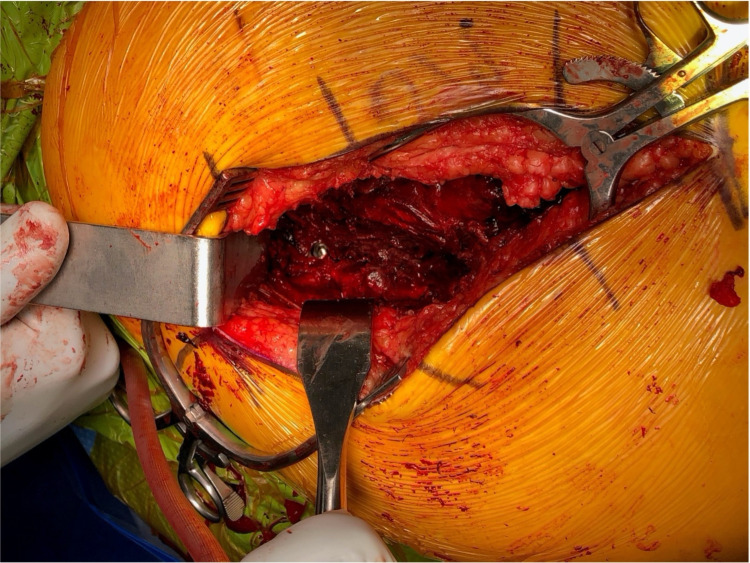
The superior gluteal artery was identified and noted to have been lacerated with active bleeding.

The patient was placed in a left lateral decubitus position, and a curved incision was made over the right posterolateral hip for a Kocher-Langenbach approach. Electrocautery dissection was taken down to the level of gluteus maximus muscle fascia. The fascia was carefully incised, and the hematoma was progressively evacuated from under the gluteus maximus and gluteus medius planes. After evacuation, the greater trochanter became palpable. Once the surgical bed was cleared of hematoma, the SGA was identified. A branch of the SGA was noted to have been lacerated with active bleeding (Figure 6). The vessel was clamped and ligated using 3-0 silk sutures. A 15-French subfascial Jackson Pratt drain was placed at the end of the procedure. On postoperative day three, he was discharged in improved condition. His surgical drain was removed at the three-week postoperative visit. At his two-month follow-up visit, the patient’s right-sided SI joint pain was gone. At four months, the patient was walking unassisted and was not requiring pain medicines.

## Discussion

Sacral dysmorphism

Sacral dysmorphism describes any abnormal anatomy of the sacrum and its relation to the lumbar spine and pelvis. Typically, lumbosacral transitional vertebrae (LSTV) are present that shares characteristics of both the lumbar and sacral vertebrae. The Castellvi classification is one common way radiologists describe the degree of transition at the lumbosacral junction, which ranges from the presence of a dysplastic transverse process on the last lumbar vertebra to complete fusion of the last lumbar vertebra to the sacrum [4]. According to this classification, our patient represented a Castellvi type 3b, having both transverse processes fused to the sacral ala. Illeez et al. demonstrated the importance of an LSTV when considering low back pain and SI joint pain. They studied 500 patients with low back pain and found the presence of LSTV in 26% of patients [5].

The prevalence of LSTV is further described by Matson et al. as a range of 3.9-35.6% in the spine literature and upwards of 50% of the population in the trauma literature. They note that the dysmorphic sacrum in this patient population results in no safe corridor for a horizontal S1 screw, rather the preferred safe zone is oblique from caudal to cranial and posterior to anterior [4]. Further recommendation includes preoperative CT evaluation of the pelvis, which has become routine at most institutions, as well as CT navigation for SI joint fusion.

Kurosawa et al. further noted the importance of LSTV as it pertains to SI joint arthrodesis. In a case report, they noted perforation of the anterior cortex with L5 nerve root injury that was not identified intraoperatively on an inlet view [6]. They recommended a lateral pelvic view to further place the most cranial S1 screw. The variation in sacral morphology cannot be overstated as noted by Mahato et al., who further demonstrated that LSTV exists as a wide spectrum [7].

In addition to LSTVs, Routt et al. found many other anatomical variations in patients with a dysmorphic sacrum to include mammillary bodies in the alar region, tongue-in-groove appearance of the SI joint, residual upper sacral disc space, collinearity of the top of the sacrum, and the iliac crests, dysmorphic sacral neural foramina that are not circular in shape, and increased alar slope that is not collinear with the iliac cortical densities on the lateral radiograph [8]. While our patient did not have all of these characteristics, the S1 body did have mammillary bodies, and there was a residual S1-S2 disc. Also, the alar cortical densities on the lateral radiograph were more caudal when projected onto the S1 body than we typically see.

Weigelt et al. performed an analysis of 269 CT scans of the pelvis to determine the prevalence of sacral dysmorphism and the possible correlation of these characteristics to the size of the SI joint surface area. Interestingly, they found that the presence of residual upper sacral discs, which was present in our patient, correlated with a significantly smaller SI joint surface area [9]. This limits the options of implant placement to a smaller confined space. The presence of the S1-S2 residual disc and lumbarization of S1 resulted in our intentional placement of implants being more caudally than we typically place them.

Potential injury to the superior gluteal artery

The SGA is a branch of the internal iliac artery that exits the true pelvis through the greater sciatic foramen where it quickly divides into superficial and deep branches, which supply the gluteus medius and gluteus minimus muscles. A study by Collinge et al. found that with iliosacral screw placement, the deep branch of the SGA was the branch that was most likely to be injured as it exits the greater sciatic foramen. This is due to the path it takes superiorly along the posterior surface of the ilium. In their study, 18% of the iliosacral screws placed in cadavers resulted in injury to the deep superior branch of the SGA [10]. Of note, placement of triangular implants as opposed to screws across the SI joint has resulted in much lower complication rates of 1.3-3.5% [3, 11, 12].

The SGA also has several anatomic variations which make pre-surgical planning crucial. Hamabe et al. looked at 81 patients using contrast-enhanced CT. In their study, they found 82% of the SGAs ran between the L5 and S1 spinal nerve branches and 17% of SGAs ran lateral to the L5 branch [13]. Interestingly, they also found patients with variations of how two internal iliac veins communicate and form a venous loop around the SGA before continuing dorsally.

Potential injury to superior gluteal nerve and associated nerve roots

Along with injury to the SGA, it is important to note potential injury to adjacent nerves and nerve roots. The superior gluteal nerve (SGN) is composed of the L4-S1 nerve roots from the sacral plexus. The SGN typically exits the greater sciatic foramen as two separate branches that follow the deep and superficial branches of the SGA [10]. The SGN leaves the greater foramen above the piriformis muscle and supplies the gluteus medius, gluteus minimus, and tensor fasciae latae muscles.

The L5 and S1 nerve roots are of special concern because the L5 nerve root is located on the anterior sacral alar surface and the S1 nerve root takes a similar course through the anterior sacral foramina. The L4 nerve root can also be at risk as it is commonly on the ventral side of the sacrum, although usually more lateral than the L5 nerve. The interosseous pathways of the L5 and S1 nerve roots are avoided by taking certain safe zones. The safe zones in patients with normal sacral anatomy is posterior to the sacral ala for the L5 root and anterior and cranial to the S1 root, but these safe zones are altered in sacral dysmorphism due to anatomic variations [14]. In patients with a dysmorphic sacrum the preferred safe zone is usually more caudal and located at the second sacral segment [14]. Unfortunately, when a more caudal site is chosen to place implants, the distance between the implants and the greater sciatic foramen is decreased, thus increasing the risk of injury to a more proximal branch of the SGA.

Literature review

Since there is a lack of literature discussing injury to SGA during minimally invasive SI joint fusion using triangular implants, an evaluation of the literature on iatrogenic SGA injuries from iliosacral screw placement was performed to determine how these injuries have been treated (Table 1). Relevant articles were found on PubMed using the search terms "superior gluteal artery" and "screw placement". In each reported injury, embolization was performed to stop the bleeding. In our patient, interventional radiology was consulted for embolization of the SGA, but they elected not to perform embolization since no active extravasation was seen on CTA of the lower extremity. Therefore, surgical exploration with successful ligation of the artery was performed due to our patient’s persistent symptomatic anemia, concern for ongoing bleeding, and painful submuscular hematoma.

**Table 1:** Iatrogenic SGA injury after iliosacral screw placement. F: Female, M: Male, SGA: Superior Gluteal Artery

Author, Year	Age, Sex	Indication for Screw Placement	Complication	Treatment for Complication	Follow-up
Altman et al., 1999 [15]	69 y.o., M	Left displaced transforaminal sacral fracture	Intraoperative SGA hemorrhage	Coil and gelfoam embolization	Discharged to nursing facility 15 days after injury.
Garín et al., 2021 [16]	83 y.o., F	U-shaped sacral fracture	Post-operative hematoma from SGA bleeding	Coil and gelatin embolization	12 months, progressively returning to baseline
Maled et al., 2007 [17]	23 y.o., F	Left displaced transforaminal sacral fracture	Pseudoaneurysm of SGA	Coil embolization	Three months, weight bearing as tolerated

## Conclusions

SI joint dysfunction is a significant contributor to low back pain, which can be improved with percutaneous SI joint fusion. This is a safe and relatively simple procedure but there is an increased risk of injury to surrounding structures when a patient has dysmorphic lumbosacral anatomy. We presented the case of a patient with sacral dysmorphism that presented with hip and low back pain that was reproducible with SI joint provocative testing. The patient underwent SI joint fusion, but he returned due to pain and difficulty ambulating. Imaging found a large gluteal hematoma with disruption of the SGA. Surgical intervention found that the SGA was lacerated, and ligation of the artery led to full recovery. 

When there is a concern of sacral dysmorphism or an abnormal course of the SGA, there are several considerations that need to be addressed. First, pre-surgical planning is essential. Additional imaging studies such as a preoperative CTA pelvis or catheter-based angiogram may be performed. Taking unsubtracted views during the angiogram of the lateral pelvis will show the SGA and bone at the same time so the course of the SGA after it exits the greater sciatic foramen can be seen. Next, modifications of the standard surgical approach may be taken to allow better visualization. If a transgluteal approach is taken, consider making a larger incision to visualize the ilium prior to placing guide pins or broaching the bony channel to ensure no vascular and/or neural structures are in the way. Alternatively, a posterolateral approach to the SI joint rather than a transgluteal approach may be performed to avoid SGA and SGN branches crossing the posterior aspect of the ilium. Regardless, patients with sacral dysmorphism must be counseled of their higher risk of injury before surgery.
